# Rapunzel Syndrome Complicated by Cholecystoduodenal Fistula Secondary to Biliary Compression: A Case Report of a Patient With Cerebral Palsy

**DOI:** 10.7759/cureus.102568

**Published:** 2026-01-29

**Authors:** José Serafio-Gómez, Ian-Arfaxad Saldaña-Badillo, Dana Karina Mauleon-Tiscareño, Juan Pablo Pérez Bucio, Angela Márquez Romero, Yozgart Aldahir Hornedo García

**Affiliations:** 1 General Surgery, Chihuahua City General Hospital “Dr. Salvador Zubirán Anchondo”, Chihuahua, MEX; 2 Medicine, Universidad Cuauhtémoc Aguascalientes, Aguascalientes, MEX; 3 College of Medicine, Universidad Cuauhtémoc Aguascalientes, Aguascalientes, MEX; 4 Medicine and Surgery, Universidad Cuauhtémoc Aguascalientes, Aguascalientes, MEX

**Keywords:** compresión biliar, fistula vesicoduodenal, hepatoyeyunostomía, paralisis cerebral infantil, sindrome de mirizzi tipo v, sx rapunzel, tricobezoar, tricofagia

## Abstract

Rapunzel syndrome is a rare form of gastric trichobezoar that extends into the small intestine, leading to intestinal obstruction. Biliary-enteric fistulas are abnormal communications between the biliary system and the gastrointestinal tract, generally occurring spontaneously. We report the case of a 26-year-old female patient with a history of cerebral palsy who presented to the emergency department with an acute abdomen. Computed tomography revealed a mass occupying the stomach and intestine. A laparotomy was performed, identifying a gastric and duodenal trichobezoar complicated by an acute perforated gastric ulcer, in addition to a vesicoduodenal fistula secondary to extrinsic compression of the gallbladder. This fistula clinically mimicked a type V Mirizzi syndrome. A Roux-en-Y hepaticojejunostomy was carried out. The patient died from nosocomial pneumonia 30 days later. This case highlights the importance of a multidisciplinary approach in patients with underlying neurological conditions and underscores the surgical feasibility of hepaticojejunostomy in complex scenarios of secondary biliary involvement.

## Introduction

Rapunzel syndrome is an extremely rare manifestation of trichobezoar, characterized by the accumulation of ingested hair in the stomach, which may extend into the small intestine and cause symptoms ranging from digestive discomfort to complications such as obstruction, ulceration, or perforation. This condition occurs predominantly in young women with a history of trichophagia (hair ingestion) and trichotillomania (compulsive hair-pulling) [1].

When a trichobezoar reaches a considerable size, it can compress adjacent organs such as the gallbladder, favoring the development of biliodigestive fistulas. Among these, the vesiculoduodenal fistula, an abnormal connection between the gallbladder and the duodenum, may clinically mimic Mirizzi syndrome type V, in which an impacted gallstone causes obstruction of the common hepatic duct along with an abnormal cholecystoenteric communication. Although Mirizzi syndrome is usually lithiasic in origin, in this case the fistula was caused by extrinsic compression from the trichobezoar, an exceedingly rare clinical finding [2].

Diagnosis is often delayed, particularly in patients with neurological or developmental disorders such as cerebral palsy, due to difficulties in expressing symptoms. This increases the risk of severe complications, including gastric perforation, necrosis, and enteric fistulas. In this regard, a recently published case in Cureus highlights the importance of a multidisciplinary approach in such patients, integrating imaging, endoscopy, surgery, and psychological management to prevent fatal outcomes [3].

This article presents the case of a 26-year-old female with cerebral palsy and a history of trichotillomania, who was admitted to the emergency department with an acute abdomen. Computed tomography revealed a gastric and intestinal mass consistent with trichobezoar. Intraoperatively, a gastric and duodenal trichobezoar was identified, complicated by a perforated gastric ulcer and a cholecystoduodenal fistula secondary to gallbladder compression. To our knowledge, this is the first reported case of a trichobezoar causing a cholecystoduodenal fistula [4].

## Case presentation

**Figure 1:**
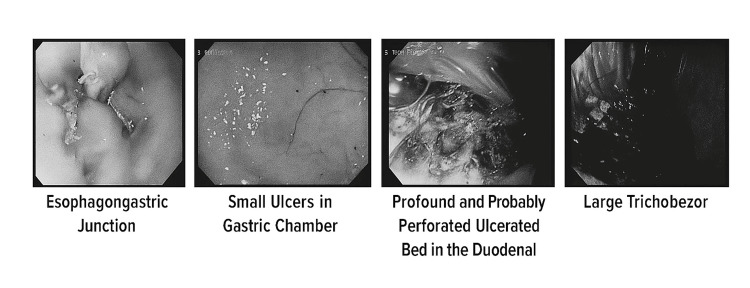
Radiologic impressions.

**Figure 2:**
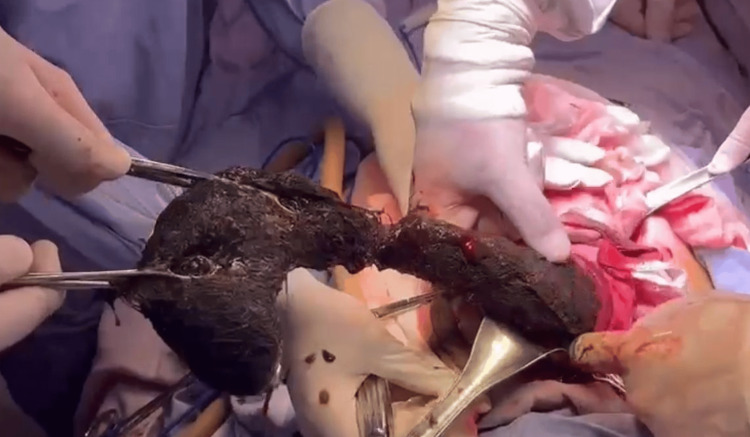
Trichobezoar removed from the gastric cavity, extending to the second portion of the duodenum. Its thickest portion measured approximately 45 cm in length.

**Figure 3:**
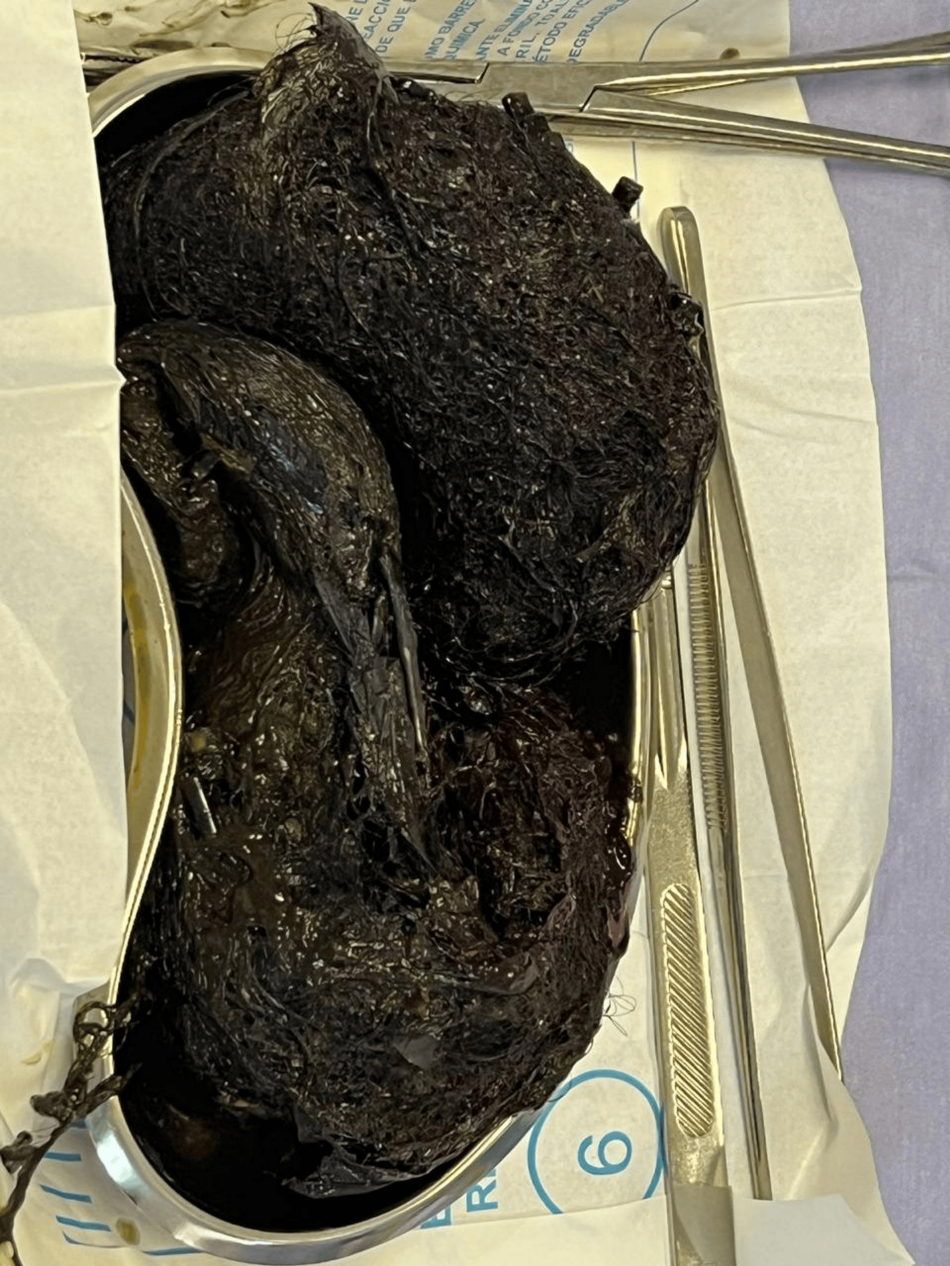
Trichobezoar removed on a kidney tray.

A 26-year-old female patient with cerebral palsy and a history of trichotillomania presented to the emergency department with an acute abdomen. A computed tomography (CT) scan revealed a mass occupying the stomach and intestine. The patient had a history of laparotomy for a gastric trichobezoar. Laboratory tests showed leukocytosis at the expense of neutrophils; therefore, surgical intervention was indicated. Intraoperatively, a gastric and duodenal trichobezoar complicated by an acute perforated gastric ulcer was found, in addition to a vesicoduodenal fistula secondary to extrinsic compression of the gallbladder by the trichobezoar. This fistula clinically mimicked a Mirizzi syndrome type V. Consequently, a biliodigestive derivation was performed. This procedure consisted of creating an anastomosis between the hepatic duct and a previously isolated jejunal loop, allowing bile drainage into the digestive tract, while intestinal continuity was restored through a more distal enteroanastomosis (Tables 1-3; Figures 1-3).

**Table 1:** Hematological laboratory results of the patient.

Parameter	Result
White blood cell count (WBC)	10.12 10^3/ul
Red blood cell count (RBC)	2.91 10^6/ul
Hemoglobin (Hb)	8.60 g/dL
Hematocrit (Hct)	26.20 %
Platelet count	562 10^3/ul
Lymphocytes	19%
Segmented neutrophils (Segs)	77%

**Table 2:** Relevant clinical chemistry laboratory results.

Parameter	Result
Direct bilirubin	1.21 mg/dL
Indirect bilirubin	0.01 mg/dL
Total bilirubin	1.22 mg/dL
Serum creatinine	0.4 mg/dL

**Table 3:** Special preoperative laboratory results.

Parameter	Result
Procalcitonin	0.62 ng/dL

No complications occurred during the surgical procedure.

In the immediate postoperative period, the patient was admitted to the intensive care unit for close monitoring and advanced management due to the complexity of the procedure and her baseline condition. During her stay, a central venous catheter was placed as part of the required supportive measures. Subsequent imaging revealed the presence of a pneumothorax; therefore, a water-seal chest tube was inserted to facilitate lung re-expansion. Initially, the drainage system appeared to function appropriately; however, on a later assessment, disruption of the seal was identified, resulting in persistence of the pneumothorax and progressive respiratory deterioration. Once the system’s integrity was restored, proper drainage was reestablished, and the necessary interventions were performed to stabilize the patient.

Despite all implemented measures and multidisciplinary support, the patient developed persistent hemodynamic instability and progressive multiorgan dysfunction over the following days. Her clinical course failed to demonstrate sustained improvement, and she ultimately suffered a cardiopulmonary arrest that did not respond to advanced resuscitative efforts, resulting in her death in the intensive care unit.

This clinical course highlights the complexity of the patient’s postoperative state and the convergence of multiple critical factors during her follow-up, which were determinants in her outcome. The case underscores the importance of continuous monitoring and systematic verification of device function in patients with high-risk postoperative conditions.

## Discussion

This case highlights the exceptional rarity of a trichobezoar causing a cholecystoduodenal fistula, a complication that clinically mimics Mirizzi syndrome. To our knowledge, no previous reports have documented a vesiculoduodenal fistula secondary to extrinsic gallbladder compression by a trichobezoar, making this presentation an unusual and clinically significant variant within the Rapunzel syndrome spectrum.

Rapunzel syndrome is characterized by a gastric trichobezoar extending into the small intestine, typically associated with trichophagia and underlying psychiatric disorders such as trichotillomania and anxiety [5]. It predominantly affects young patients and often presents with nonspecific abdominal symptoms until complications arise. In this case, abdominal CT demonstrated a large gastric and duodenal trichobezoar, and surgical exploration revealed an acute perforated gastric ulcer with a secondary cholecystoduodenal fistula produced by mechanical compression from the bezoar. Due to the extent of biliary involvement and the inability to perform direct repair, a Roux-en-Y hepaticojejunostomy was required [6].

Although bilioenteric fistulas may occur in gallstone-related disease, their development as a consequence of a trichobezoar is exceedingly rare. The mass effect of the bezoar at the duodenal level likely contributed to ischemia, inflammation, and subsequent fistula formation, reproducing the clinical behavior of advanced Mirizzi syndrome (type V).

A crucial component of this case is the psychiatric and neurodevelopmental background of the patient. Cerebral palsy is strongly associated with a higher prevalence of psychiatric disorders, particularly anxiety, reported in up to 62% of patients. Anxiety-driven trichotillomania can progress to trichophagia, leading to trichobezoar formation and, in severe cases, complex gastrointestinal and biliary disorders [7]. These behaviors may be influenced by the neuropsychological and emotional consequences of cerebral palsy [8]. In this context, the patient’s history is consistent with the established relationship between cerebral palsy → anxiety → trichotillomania/trichophagia → bezoar → complications, reinforcing previously described associations [9].

The convergence of cerebral palsy, psychiatric comorbidity, and a severe abdominal complication underscores the importance of multidisciplinary evaluation, early recognition of high-risk behaviors, and integrated medical-psychiatric management. This case highlights the need to consider trichobezoar-related complications in neuropsychiatric patients presenting with acute abdomen, as timely diagnosis and surgical intervention remain crucial to preventing life-threatening outcomes.

## Conclusions

This case highlights an exceptionally rare and complex presentation of Rapunzel syndrome, in which a gastroduodenal trichobezoar caused extrinsic compression and led to a vesiculoduodenal fistula in a patient with cerebral palsy. This unusual association underscores the importance of maintaining a high index of suspicion for abdominal complications in patients with neurological impairment and behavioral disorders such as trichophagia and trichotillomania. The clinical picture closely resembled advanced Mirizzi syndrome, emphasizing the need for thorough diagnostic and surgical assessment. Ultimately, successful management with a Roux-en-Y hepaticojejunostomy demonstrates the effectiveness of this approach in complex cases involving significant biliary involvement and reinforces the life-threatening potential of abdominal manifestations of trichotillomania. 
